# Prevalence of personality disorder diagnoses in people referred to specialized gender identity clinics in Finland

**DOI:** 10.1192/j.eurpsy.2025.753

**Published:** 2025-08-26

**Authors:** M. E. Kaila-Vanhatalo, T. Tolmunen, A. Mattila, R. Kaltiala

**Affiliations:** 1 Faculty of Medicine and Health Technology, Tampere University, Tampere; 2 Department of Adolescent Psychiatry, Oulu University Hospital, Oulu; 3 Department of Adolescent Psychiatry, Institute of Clinical Medicine, University of Eastern Finland, Kuopio; 4 Division of Psychiatry; 5 Department of Adolescent Psychiatry, Tampere University Hospital, Tampere; 6 Vanha Vaasa Hospital, Vaasa, Finland

## Abstract

**Introduction:**

Personality disorders are defined as maladaptive traits of personality and behavior causing severe harm to an individual or people around them. Personality development is closely related to identity development. Higher prevalence on personality disorder diagnoses has been found to be associated with gender dysphoria. However, previous research on this topic has been scarce and methodically varying (Table 1, Image 1&2).

**Objectives:**

The object of this research was to determine the prevalence of personality disorder diagnoses in individuals requesting medical gender reassignment.

**Methods:**

A register-based follow-up study of individuals who contacted the nationally centralized gender identity services in Finland in the period 1996-2019 (n=3,665) and 8:1 age and sex-matched population controls (n=29,292). All their specialist-level psychiatric treatment contacts in 1994-2022 were identified in the National Care Register for Health Care. ICD-10 diagnoses and dates of the contacts were extracted.

**Results:**

Among the gender dysphoria group, 15.0% (551 out of 3,665) had received a diagnosis in the personality disorder group (F60-69 excluding F64.x) in specialized psychiatric health care, while among the control subjects, 2.1% (625 out of 29,292) had received such a diagnosis (Table 2). The most common personality disorder among the gender dysphoria group was borderline personality disorder.

**Image 1:**

**Figure:**
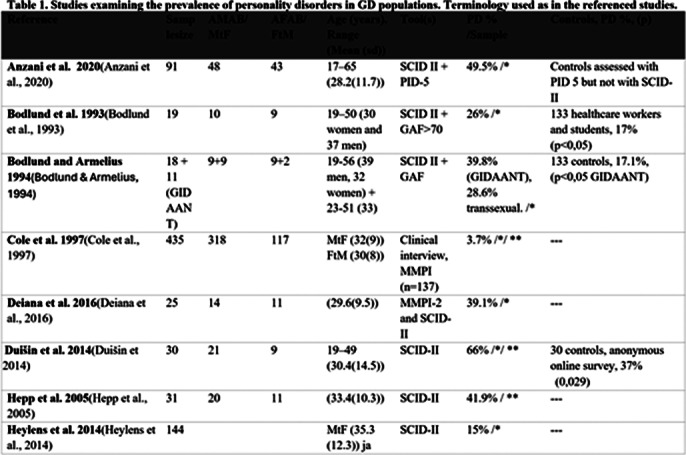

**Image 2:**

**Figure:**
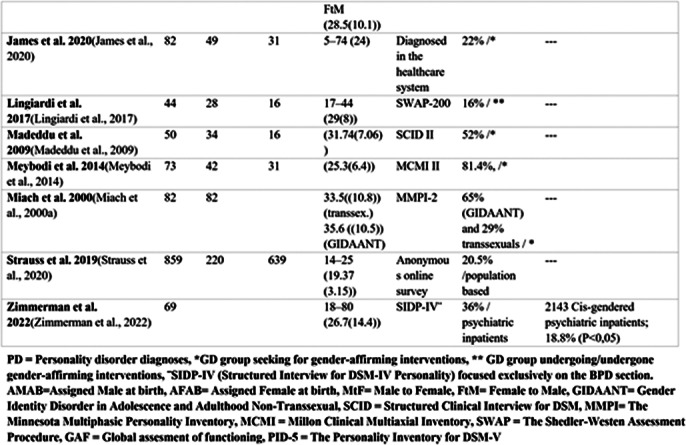

**Image 3:**

**Figure:**
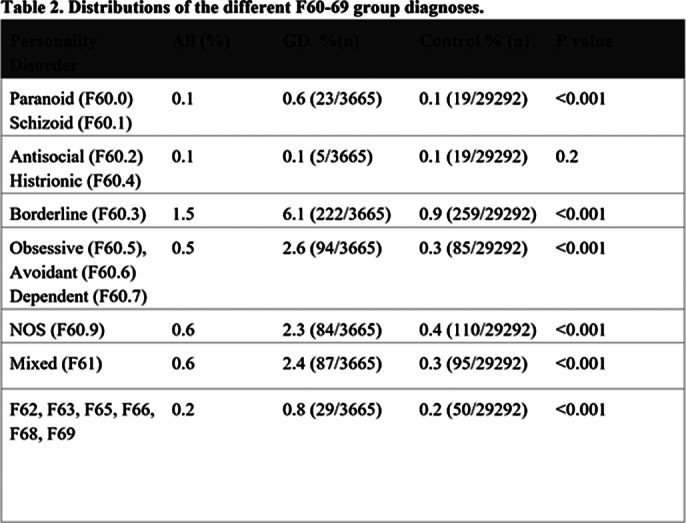

**Conclusions:**

Personality disorders are overrepresented among those seeking medical gender reassignment. Further research is needed to explore the factors contributing to the higher prevalence of personality disorders among individuals with gender dysphoria.

**Disclosure of Interest:**

None Declared

